# Reports on the Hospitals of the United Kingdom

**Published:** 1910-01-22

**Authors:** Henry Burdett


### REPORTS
ON THE
### Hospitals of the United Kingdom.

By SIR HENRY BURDETT, K.C.B., K.C.V.O.

### XXI.—ROYAL SOUTH HANTS AND SOUTHAMPTON HOSPITAL.

Seventeen years have passed since we first inspected this institution at a time when the dawn of its present prosperity had barely reached Southampton. In those days the greater part of the hospital buildings were old and out of date, and were ripe for demolition. The hospital possessed no quarters for its nursing staff, but a new out-patient department had just been completed and opened. Several of the older wards were overcrowded, and the infirmary as it then existed was altogether unworthy of the town of Southampton.

### Then and Now.

We called attention to these defects, and within a few years the old buildings were demolished and new and improved wards with every unit perfected and up to date were provided. Indeed, the plan of reconstruction was so good that many experts visited the institution, and some of its features now enjoy the flattery of imitation by being reproduced in some of the best modern hospitals in this country and abroad.

Some ten years or more have elapsed during which the institution has been efficiently worked and administered in all its departments. Sufficient time has passed to test much of the work put into the newer portions of the hospital, a fact which lends additional interest to our inspection. Unfortunately, the matron, Miss Mollett, was away on the day of our visit, and that was a great loss in every sense, because she has been identified with the re-organisation and new features of management which have greatly added to the reputation of this institution. Besides, Miss Mollett is one of the ablest of provincial matrons.

### Attend to Repairs and Maintenance.

It is not unusual to find that when an institution that has fallen behind is brought up to a high standard of efficiency, the tendency is to consider everything so new and complete that the management gets out of the habit of paying full attention to repairs and maintenance every year. So appliances, fittings, and even internal decorations and upkeep may be allowed to retrograde until the institution is once again face to face with the necessity for a large expenditure. It seemed to us that unless prompt measures are taken to renew certain of the fittings, and generally to take up actively the question of repairs and maintenance, this institution will before long lose much of the interest it formerly excited. The slop sinks have not worn well. Much of the enamelling has perished, and we would counsel the managers to call the attention of the architects and the makers to these defects, and also to the same defect in one of the baths, with a view to the re-enamelling or renewal of these articles upon special terms, whereby the cost is in part at least borne by the makers. We have seldom seen any apparatus of the kind anywhere which has worn so badly as that supplied to this hospital. Every visitor who knows his business must have his attention drawn to these defective fittings, which must be a source of annoyance to Messrs. Young and Hall, who scheduled these fittings and had them fixed at Southampton. Where exactly the fault lies we were unable to ascertain, but the managers should promptly look into the matter and have the necessary renewals effected without loss of time. We would recommend the appointment of a special committee to inspect carefully the whole of the buildings, with the object of bringing everything up to the highest pitch of efficiency. Steps should be taken to secure the more active and continuous inspection of every part of the hospital at regular intervals.

### A Disappointing Laundry.

We were particularly struck on visiting the laundry by the want of plan and system which these relatively new buildings exhibit. A laundry building should be so arranged as to provide for the easy circulation of all articles sent to the wash. It should make certain that all foul and soiled articles are received, kept, and dealt with separately; arrangements should be possible whereby all the staff linen can be kept absolutely apart and distinct. Judged by these essentials, the buildings in question are distinctly not up to date, they must be unnecessarily troublesome to work, and they should be reconstructed and made much more efficient than they are at present. It is surprising that a hospital which contains so many good points structurally should be saddled with so indifferent a laundry of such recent construction.

### Slop Sinks.

Another relatively small but important matter is the slop sinks throughout the hospital. The wings or strainers of these sinks are made of teak, and they nearly always present the appearance of being ill-kept and unclean. In fact, however much care and labour may be expended upon them, teak sinks never have and never will answer for hospital purposes. Even slate strainers are better than teak, but block tin are the best for hospital purposes. There are also a number of good earthenware patterns now available from which a selection could be made. The old idea that stone and earthenware sinks contribute to breakage has been disproved by the staff of every well-managed hospital of to-day.

Making every allowance for the wonderful improvements which have been effected in this hospital during the last fifteen years, paying a high meed of praise to the operation theatre, which is good, a general survey of the whole establishment as we found it on the day of our visit leads us to the conclusion that there is a distinct need that the managers should arouse themselves and wake up. As already pointed out, money should be voted at once for repairs and maintenance account, and we hope that the public and those more immediately interested in the hospital itself will show more liberality. Money is wanted for a number of relatively little things, and the matron should be liberally treated and given a free hand in regard to them. We consider, for instance, that more chairs and comfortable furniture should be supplied to the nurses' sitting-room. In our view such furniture should err, if it err at all, on the side of comfort and attractiveness.

Features of Special Interest.

One or two features of especial interest attach to the older buildings. Thus a tablet recording a gift to a popular member of the honorary medical staff who retired upwards of thirty years ago commences with the words: "The porter, nurses and servants of the Royal South Hants Infirmary in testimony, etc." This tablet amusingly records the relative positions of the minor officials in this hospital at the period in question. We can imagine the indignation with which such a record would be received at the present day. The hospital also contains the most interesting specimens of the peephole system which Miss Nightingale used to make such a point of. Nowadays in every well-administered hospital when a sister or nurse is on duty, she has to keep at her work all the time. When her hours of duty are over she is protected from the peephole worry by being replaced by an able substitute, and has her room well away from the ward. The children's ward is evidently in the hands of an able sister who knows her work and does it. She it was, we presume, who thought out and procured the little round table and armchairs of various patterns at which the children here sit and have their meals. This constitutes the most attractive feature in a children's ward which we have ever seen.

---

**The General Infirmary, Leeds,** will be reported on next week.

